# SLE classification criteria: Science-based icons or algorithmic distractions – an intellectually demanding dilemma

**DOI:** 10.3389/fimmu.2022.1011591

**Published:** 2022-09-28

**Authors:** Ole Petter Rekvig

**Affiliations:** ^1^ Fürst Medical Laboratory, Oslo, Norway; ^2^ Department of Medical Biology, Faculty of Health Sciences, UiT The Arctic University of Norway, Tromsø, Norway

**Keywords:** systemic lupus erythematosus, SLE classification criteria, incongruent SLE cohorts, monogenic SLE, quasi SLE diagnostic criteria

## Abstract

It is, so to say, not a prerogative authority assigned to SLE classification criteria that allow them to declare something definitively important about SLE. This is particularly true as criteria-based classification processes overrule the highly needed evolution of concise diagnostic criteria. It is classification criteria that allocate SLE patients into cohorts intended to describe the nature of their disease. Therefore, all major SLE classification criteria since the 1971 preliminary criteria usurp the role of diagnostic criteria. Today´s practice silently accept that the SLE classification process “diagnose” SLE patients despite the fact that classification criteria are not accepted as diagnostic criteria! This is a central paradox in contemporary SLE research strategies. Contemporary SLE cohorts are designed to investigate SLE´s etiological features. However, each cohort that is categorized by classification criteria has one central inherent problem. From theoretical and practical arguments, they embody multiple distinct clinical phenotypes. This raises the critical and principal question if phenotypically heterogenic SLE cohorts are useful to identify basic SLE-specific etiology(ies) and disease process(es). In times to come, we must prioritize development of firm diagnostic criteria for SLE, as the classification criteria have not contributed to reduce the enigmatic character of the syndrome. No radical improvements are visible in the horizon that may lead to concise investigations of SLE in well-defined homogenous SLE cohorts. We must develop new strategies where studies of phenotypically standardized cohorts of SLE must be central elements. Problems related to contemporary SLE classification criteria are contemplated, analyzed, and critically discussed in this study.

## Introduction

SLE classification criteria have a strong impact on SLE research. They have promoted new hypotheses and raised important question as to the fundamental nature of the syndrome. The SLE classification criteria have, on the other hand, negative consequences for central research hypotheses. A discussion of these consequences represents the center of attention for this study.

SLE is an enigmatic complex disease difficult to comprehend by rational measures (1–4). If SLE is one disease, or a cluster of related unique syndromes is still controversial (4–7). The diversity of clinical phenotypes that constitute criteria-based SLE cohorts argue for the viewpoint that SLE is likely more plural than one disease entity: The theoretical diversity of SLE phenotypes in an SLE cohort is substantial (see arguments and calculations below). Such cohorts are regarded important as fundaments for studies of central aspects of etiology and pathogenesis that cause - or aggravate - SLE, and to test experimental therapeutic strategies (8).

In contrast to these scientific strategies, an alternative critical viewpoint is that contemporary classification criteria define cohorts of SLE patients presenting a diversity of clinical phenotypes [discussed in (4, 9)]. This fact is not in conformity with, and argue against the statement that the unremitting refinements of SLE classification criteria define patient cohorts suitable for insightful and basic studies of SLE (10). If the rules are strictly followed, a classified cohort is inevitably established with patients that differ in organ involvement, autoimmune responses, severity, course and in genetic composition.

New strategies, and profound reconsiderations of the impact of SLE classification criteria are imperative to obtain an alternative science-based insight into SLE-related diagnostics, classification, etiology and pathogenesis. This is what this study deals with.

### “The one duty we owe to history is to rewrite it”

This citation [Oscar Wilde (11)] is relevant, actualized over time and in this study applied to the history of the steadily increasing numbers and categories of SLE classification criteria. The need to rewrite this history is funded on the concrete conflict between the aims defined for implementation of the criteria, and the factual heterogeneity of cohorts established as a consequence of these criteria.

SLE classification criteria operate as entry criteria for SLE cohorts aimed to investigate fundamental aspects of SLE, and many of the deviating clinical and laboratory parameters are today defined as classification criteria [see (10, 12–16)]. Importantly, the criteria do not by themselves have a common etiology and they are not linked in a pathophysiological context – at least not yet determined scientifically, nor discussed in central studies (2–4). One underscored problem is that the classification criteria are accepted irrespective whether they appear simultaneously, or individually one by one over non-defined time lapses (14).

This means that there is a silent acceptance in the relevant scientific organizations that the criteria are inconsistent with respect to their declared aims because *i.* the criteria do not represent an integrated response to a “one basic stimulus”, and *ii.* according to the rules, the criteria do not promote homogeneous and pathologically understandable SLE cohorts. From a critical point of view, we must rather consider whether we have to recreate and restructure studies of SLE cohorts defined by clusters of clearly *inter*-dependent criteria (like anti-dsDNA antibodies, lupus nephritis (17–19) and/or lupus dermatitis (20, 21) or brain disease (22–24), see below). This suggestion is in clear contrast to today´s use of a wide specter of disparate criteria that have evolved over the last 50 years (10, 12, 14–16). In this author´s contemplation, it is not the SLE classification criteria that is wrong, it is the authoritative rules for their use that is wrong. In this context, the citation of Oscar Wilde is highly relevant. Clear arguments for a revision of the status of SLE classification criteria are discussed in the following sections.

## Back to basics: Formal definition of the term criterion – and its impact on SLE classification and diagnostic

The term *criterion* conveys different propositions. One defines the essential meaning of criterion as “*something that is used as a reason for making a judgment or decision*”^1^. For the purpose of discussing criterion in the actual context, the terms “*distinguish*” and “i*dentification*” can be applied as synonymous terms *(Greek:* κρῐτήρ (*kritḗr*, ”interpreter”). However, this information relates to *one* criterion, not to clusters of differently segregated criteria that is relevant for interpreting the literature related to classification of the syndrome SLE. The use of the terms “distinguish” and “identification” are problematic when we consider the complex implementation of clusters of classification criteria in individual SLE patients.

Importantly, there is no formally accepted single criterion that classify SLE. The closest to this is defined in the SLICC classification criteria (16) that recommend that lupus nephritis in presence of ANA (irrespective specificity)? or anti-dsDNA antibodies is sufficient to classify a patient as having SLE. In that context, the two criteria serve as both diagnostic and classification criteria. A similar impact of the two criteria is provided by the 2019 EULAR/ACR SLE classification criteria (see below).

Each of the commonly used classification versions that have appeared from 1971 till 2019 identify clinical SLE phenotypes^2^ that differ from each other within a cohort with respect to autoimmunity, severity and organ involvement (2–4, 25). This is surprisingly not considered important to principally discuss in central trend-setting articles (10, 12–16). Quite contrary, SLE cohorts are implicitly defining SLE as a disease entity, a term that is explained as “*… something that has separate and distinct existence and objective or conceptual reality”* ^3^. It is also in the classification rules required that “*It is critical to strictly keep to the attribution rule, that items are only counted if there is no more likely alternative explanation than SLE*” [as stated and discussed in different published versions by Aringer et al. in e.g (10, 26, 27)]. However, the essential meaning of the term “alternative” is not discussed. The latter statement declares that SLE is a distinct disease different from alternative conditions, i.e. a disease that is recognizable in a differential diagnostic context. Here, it is a remarkable factual overlap between the confusing definition of “*SLE*” and the vague and open term “*alternative*”. The statement expressed by Aringer et al. may therefore not be valid as a normative instruction with impact on analytical studies and scientific discussions.

### SLE classification criteria: Science-based algorithms or imprecise approximations?

*“An algorithm in a general context means a systematic procedure that produces — in a restricted number of steps — the answer to a question or the solution of a problem”^4^. *In our context, SLE classification criteria are assumed to operate as algorithms aimed to classify the syndrome and to describe its basic aspects. In what sense can widely diversified algorithms like classification criteria (see details in **Table 1**) help solving enigmas linked to a complex syndrome like SLE? It may be fair to underline that we still lack a firm rational and evidence-based link between the basic SLE-promoting process(es) and the involvement and impact of a number of SLE classification criteria.

**Table 1:** Comparison* of SLE classification criteria in 4 different classification versions from 1971-2019**.

1971 preliminary SLE Classification criteria	1982 ACR SLE Classification criteria	2012 SLICC SLE Classification criteria	2019 EULAR/ACR SLE Classification criteria^#^
1.Facial erythema (butterfly rash) 2.Discoid lupus erythematosus 3.**Raynaud phenomenon** **4. Alopecia** 5.**Photosensitivity** 6.Oral or nasopharyngeal ulceration 7.Arthritis without deformity 8.**Lupus erythematosus cells** 9.**Chronic false-positive serologic test for syphilis** 10.Profuse proteinuria 11.**Cellular casts** 12.Pleuritis or pericarditis 13.Psychosis or convulsions 14.Hemolytic anemia or leukopenia or thrombocytopenia	1. Malar rash 2. Discoid rash 3. **Photosensitivity** 4. Oral ulcers 5. Synovitis 6. Serositis 7. Neurologic manifestations 8. Renal manifestations 9. Hematologic manifestations 10. **Immunologic manifestations:** **Anti-DNA/Anti-Sm** **antibodies** Anti-phospholipid antibodies* 11. **ANA**	Clinical Criteria: 1. Acute cutaneous lupus 2. Chronic cutaneous lupus 3. Oral ulcers: palate 4. **Nonscarring alopecia** 5. Synovitis involving two or more joints or tenderness in two or more joints 6. Serositis 7. Renal disorder 8. Neurologic disorder 9. Hemolytic anemia 10. Leukopenia (< 4000/mm3 at least once) 11. Thrombocytopenia (<100,000/mm3) at least once **Immunological Criteria:** 1. **ANA above laboratory reference range** 2. **Anti-dsDNA above laboratory reference range** 3. **Anti-Sm** 4. Antiphospholipid antibodies* 5. **Low complement** 6. Direct Coombs test	**Obligatory Entry criterion Antinuclear antibodies** 1.**Constituional fever** 2.Acute cutaneous lupus 3.Subacute cutaneous OR Discoid lupus 4.Oral ulcers 5.**Non-scarring alopecia** 6.**Joint involvement** 7.Pleural or pericardial effusion 8.Acute pericarditis 9.Proteinuria > 0.5g/24h 10.Renal biopsy class II OR V lupus nephritis 11.Renal biopsy class III OR IV lupus nephritis 12.Delirium 13.Seizure 14.Psychosis/delirium 15.Autoimmune hemolysis 16.Leukopenia 17.Thrombocyopenia 18.**Anti-dsDNA antibodies** 19.**Anti-Sm antibodies** 20.**Anti-Cardiolipin OR** **Anti-ß2GPI OR Lupus anticoagulant** 21.**Low C3 OR low C4 Low C3 and Low C4**


*This Table demonstrate a comparison between the 4 major SLE classification criteria that appeared from 1971 till 2019. In this table, only criteria without comments or weighted values are given.

**Color code:

• Criteria written in blue are unique for the only classification system they are listed under.

• Criteria written in brown are shared by some (mostly the 1982, 2012, 2019, but not all criteria sets.

• Those criteria written in black are shared by all 4 criteria sets. Criteria may here be designated differently although they express the same. For example “Renal manifestations, criterion # 8 in the 1982 ACR criteria, is in the 2012 SLICC criteria designated Renal, criterion # 7, and in the EULAR/ACR criteria denoted Proteinuria > 0.5g/24h (criterion # 9), Renal biopsy class II OR V lupus nephritis (Criterion # 10), and Renal biopsy class III OR IV lupus nephritis (Criterion # 11). These versions of criteria contain many of the same individual classification criteria and are differently annotated. These differences reflect increased insight into each criterion, and thereby different annotations, and they express the same contextual meaning.

^#^In the EULA/ACR SLE classification criteria presented in this Table, only criteria are given. For Domains see (10).

In his study “Algorithmic Chaos”, Paul Vitanyi  (28) discusses the conceptual tension between determinism and randomness (28). Ideally, hypothesis-based theories - in our context impact of criteria thought to reflect SLE - are abstract representations for the underlying physical reality, i.e. SLE as an identifiable and explainable syndrome. The title of this study refers to the following contemplation: Scientists postulate an informal description of SLE which is intuitively acceptable, here a defense for the theoretical reality contained in the abstract terms *criterion* and *syndrome*, and “*subsequently formulate one or more mathematical theories to describe the phenomena*” (28). These considerations are directly transferable to contemporary approaches attempting to understand what SLE is – and is not!

We intuitively simplify our algorithmic approach to consider SLE as one rational and concrete disease (regarded as a disease entity, see below) in contrast to be a “collection of abstract syndromes”^5^. It is legal to use the idiom “abstract” here, because SLE is symbolized by terms used for a well-defined disease (or syndrome) while SLE in fact is clinically poly-phenotypic, incoherent and incomprehensible.

The diversity of SLE conditions within an SLE cohort is principally determined by a minimum required number of observed criteria out of the total amount of criteria defined in the different published versions of classification criteria. For example, according to the rule prescribed for the ACR SLE classification criteria (14) fulfillment of a minimum of 4 criteria out of a total of 11 is required to classify a patient as having SLE.

According to the formula:


(n,r)=(n/r)=n!/(r!(n−r))!=?

the total number of combinations (each combination defines one clinical phenotype) is 330. Notably, the criteria defined by the 1982 ACR SLE classification criteria has the lowest theoretical number of clinical SLE phenotypes among the recent SLE classification criteria versions (see below). It is a problematic comprehension to realize that the 1971 preliminary classification criteria theoretically open for 1001 individual disparate clinical phenotypes using the formula given above, as 4 out of 14 criteria must be documented. This was not problematized in the presentation of this first version of the SLE classification criteria (12, 13). How can all these clusters of criteria integrate SLE into the idiom “a single disease entity” – and what does the idiom “disease entity” factually signify? Another dilemma that has not been problematized in the relevant literature is whether the increase in the number of classification criteria and clinical phenotypes after the 1982 ACR criteria set has the inevitable consequence that the prevalence of SLE in the population escalates.

### Do SLE classification criteria identify SLE as one disease entity – and is it a problem to use the term “entity” in this context?

In their recent studies, Aringer et al. discuss if SLE classified by the 2019 EULAR/ACR criteria constitutes one disease entity. In one of the reports (29) they express the following opinion: “*In fact, an association study within the EULAR/ACR classification criteria project found associations between manifestations only within organ domains. This independency of various organ manifestations argues for SLE as one disease entity*”. The validity of this conclusion depends on how they define the term “disease entity”. This is not problematized for cohorts established by the 2019 EULAR/ACR or previous versions of the SLE classification criteria.

The denotation of the term disease entity is problematized in Hucklenbroich´s *“Disease Entity as the Key Theoretical Concept of Medicine”* (30). Hucklenbroich states that “*It is the concept of disease entity that is of key importance for understanding medical pathology and theory of disease”.* It is further proclaimed that “disease entity” is strictly connected to *the concept of pathologicity.* The term “entity” is restricted and conceptually linked to “*a defined disease process or a causal etiology”*. In context of this discussion, Hucklenbroich stress that “Disease entity” is a theoretical concept of medical science, exemplified by “*the concept of particle, elementary particle, and field (in physics), or of element and compound (in chemistry)*”. “Disease entity” is theoretically not definable by purely empirical, observational terms. This contrasts the interpretation promoted above by Aringer et al. (29), that “*independency of various organ manifestations argues for SLE as one disease entity*”. Here, they do not consider or problematize the basic pathophysiological processes that account for the “organ manifestations”.

From thoughts and contemplations presented by Hucklenbroich (30), and based on interpretations given above, it is not acceptable to define SLE, as established by classification criteria, as an example of “one disease entity”. Rather, the contemporary SLE classification criteria collect phenotypically disparate disease variants into one cohort. These variants are not uniformly linked through a defined disease process or a causal etiology. Some of these variants may be distant from each other if we consider that they are defined by a low number of classification criteria among many. See in this context also a relevant discussion by S. Chatterjee (31).

### Can SLE according to the classification criteria rules be regarded as one disease with one etiology, and with one emerging clinical phenotype?

An SLE phenotype with lupus nephritis and anti-DNA antibodies is dramatically different from SLE without lupus nephritis [discussed in (19, 25)]. The same relates to SLE phenotypes with and without anti-dsDNA antibodies as analyzed by Conti et al. (32). There are many imperative reasons to further problematize the validity of this concept.

The pioneering 1971 SLE preliminary classification criteria significantly influenced the subsequent refinements of the SLE classification criteria [see **Table 1** (10, 14–16)]. In this way, the 1971 criteria served as “archetypical” algorithms aimed to reach a more reliable insight into basic aspects of the SLE syndrome. However, a distinction between assumedly primary SLE-related processes on one side and unpredictable secondary complications on the other were not discussed by Cohen et al. in their original publication (12), and not in their status report one year later (13).

Studies to formally establish SLE cohorts based on expert-based consensus (Delphi panel-like) methods was already described for the 1971 preliminary SLE classification criteria (see methodologies in (10, 12, 14–16), discussed in (4, 25, 33)). This is an authoritative approach difficult to control, because consensus practically means agreement, but the basis for the underlying essential premises were not systematically reported. How broad or strict the agreement embraces possible criteria has therefore not been problematized in the relevant literature, but may be important to define whether SLE classification criteria define a dominating “SLE-specific” phenotype or not.

When we consider orthodox SLE classification criteria, it is quite evident that they operate simultaneously or separately over time in different organ systems, and each criterion counts irrespective which SLE phenotype they are part of. If we adhere to the definition of a phenotype given above, both a single SLE patient, and a group of patients belonging to an SLE cohort, may demonstrate unique and disparate clusters of criteria that are not compatible with one exclusive SLE diagnosis. This means that a given criterion is not reflecting basic process(es) compatible with an etiology of SLE. The starting point(s) of SLE in a patient classified as having SLE is still far from being settled. This is also a reflection articulated by Aringer et al.: *“….items are only counted if there is no more likely alternative explanation than SLE*” (26, 27).

## How do we decipher impact of unequal clusters of classification criteria?

A basic problem is to understand why SLE classification criteria are so disseminated, so biologically and etiologically incoherent, and - as defined in the classification criteria – so clustered (more than one criterion is required to classify SLE). No studies on generation of SLE classification criteria have so far envisaged *why* clusters of biologically incoherent criteria are claimed to classify SLE, and why they appear clustered at all [ (10, 12, 14–16), discussed in (4, 25)].

The rational basis for these reflections is to prepare for new critical discussions relevant to promote alternative selections of criteria and their implementation in SLE research. It is vital to investigate if the criteria contribute to increased insight into the syndrome SLE – or if results of investigating SLE cohorts may potentially *reduce* the significance of such studies. The reason for this pessimistic viewpoint would not have been valid if classification criteria substantiated homogenous SLE cohorts rather than cohorts presenting multiple disparate clinical phenotypes.

For example, one question is whether SLE classification criteria can be used to basically define SLE as “the prototypic autoimmune syndrome” (see e.g. Stojan and Petri (34)). This statement depends on whether autoimmunity is defined or scientifically described as a primarily etiologic factor. The opposite argumentation is that autoimmunity is defined as a component secondary to a basic etiological defect^7^ (like functional loss of renal DNase 1 (35–38), or DNase 1L3 (39–42)), or secondary to infections or malignancies, all conditions reported to complicate SLE by promoting e.g. chromatin autoimmunity (reviewed in (4, 25, 43–45) and references therein). If then, autoimmunity is a response to an underlying SLE-promoting process, and is therefore a secondary process not causing the syndrome, *but modifying it*. This is not a purely semantic discussion, but reflects a need for definitions required to generate new or alternative hypothesis-driven research strategies. In this context, hypothetically diagnostic criteria ideally linked to a basic etiology would be advantageous.

### Classification criteria serve as quasi diagnostic criteria due to a conflict between our immature insight into SLE etiology and selection of criteria classifying it

SLE classification criteria are, whether we like it or not, used as quasi diagnostic criteria with inadequate scientific justifications allowing them to serve this purpose. This comprehension defines the theoretical conflict between SLE as an enigmatic disease confused by non-categorical criteria on one side, and a potential SLE with clear diagnostic markers linked to specific pathobiological processes on the other.

On the other side, single elements assumed to be central in SLE, like lupus nephritis (19, 46), origin and impact of anti-dsDNA antibodies (19, 45, 47), skin affections (20, 48) or cerebral lupus (22, 49, 50) are for decades studied in detail and have provided important insight into their individual pathophysiological origins (25, 51, 52). Today, we do not know the links between these concrete affections (or criteria in a wider sense) and SLE.

In their published discussion Aringer et al. declare that the 2019 EULAR/ACR classification criteria are meant for classification, and not for defining diagnostis (29). On the other hand, they argue for a (relative)? diagnostic impact of individual criteria (29). This demonstrates that there is a theoretical more than a pathobiological-based conflict between classification and diagnostics.

### SLE diagnostic criteria – may they originate from pathogenic events that are regarded unique for SLE?

It may be difficult to understand why studies on incoherent SLE classification criteria have been prioritized over the last 50 years (10, 12–16), while diagnostic criteria are still stupefied in practical medicine and in medical science [discussed in (4, 25, 29, 33)]. In practice, *we classify a disease we by cogent means are not able to diagnose* beyond using “democratic” processes like statistics, and Delphi panels consisting of highly recognized experts (see below (53, 54),).

For the first, it is difficult to propose diagnostic criteria wen we don’t agree what SLE is. In contemporary evolution of SLE classification criteria a large number of possible criteria are initially selected, and central criteria are nominated and elected by “democratic” principles (like Delphi panels) (10, 12–16). This may mean that the criteria are not required to be coherent and biologically linked in a causal relationship, and may therefore not be linked through defined pathophysiological pathways. In this context the criteria cannot diagnose SLE, and should not classify SLE due to the emerging disparate and poly-phenotypic SLE cohorts.

Based on an alternative strategy, we can from the comprehensive literature on SLE extract one criterium that is regarded a central “quintessential” (47) biomarker for SLE – the anti-dsDNA antibody. This antibody may be a clinical epiphenomenon when it does not induce inflammatory responses, but may be transformed into a pathogenic factor if chromatin fragments are exposed in e.g. glomeruli due to loss of renal DNase 1 enzyme activity (discussed in (19)). In the latter pathogenic context, the antibody may represent an origin for series of pathophysiological recognizable deviations of clinical parameters, like organ affections and inflammatory parameters (discussed in 4;19).

In this context, cohorts classified (solely) by pathogenic anti-dsDNA antibodies may allow description of consequent evolving organ affections and biological disease parameters that may turn out to be linked to the pathophysiological effects of these antibodies. This approach has not been investigated systematically. In this way, patients that differ in criteria combinations (like with or without anti-dsDNA antibodies and with or without lupus nephritis) will appear phenotypically different, and will receive different diagnostic description (discussed in (9, 19, 20).

## SLE classification criteria: Evolution, impact and limitations

It is not the SLE classification criteria that are wrong, but the authoritative rules that determine the use of them. The SLE classification criteria are considered to “*define the patient population for clinical trials and translational studies, but also to influence current understanding of the disease*” (53).

These two statements are problematic to conceive. Contemporary SLE classification criteria factually define a large group of pathobiologically different patients, and, for example, autoimmunity to DNA does not predict inflammation in a given SLE patient ( (4, 25), discussed in (18)). Therefore, it is questionable if an SLE cohort that is selected based on presence of few criteria among many possible ones is useful for clinical trials or to influence our current understanding of the syndrome. The basis for such clinical and experimental approaches requires in this author`s opinion, cohorts of clinically, or immunologically/biologically homogenous SLE patients.

The number of criteria is growing in the refined classification criteria versions although critical reflections related to their concise impact on composition of SLE cohorts are not easy to observe (see e.g (26, 54).). Schmajuk et al. performed rounds of Delphi exercises involving a large international group of expert lupus clinicians to select SLE classification criteria. They provided a broadly representative view of the current insight into classification of SLE. A set of 40 candidate criteria for the classification of SLE for further evaluations were identified in that process (54). This process is based on a “democratic” election process voting for or against possibly valid criteria.

An alternative principal discussion was published by Dougados and Gossec. They discussed strategies beyond Delphi panels related to *why linked to how* classification criteria may be generated (55).

In contrast to the fact that the 2019 EULAR/ACR SLE classification criteria does not focus on inherent adverse aspects of the criteria versions (see e.g (8, 27, 29, 56)., Infantino et al. point at possible undesirable implications of the classification criteria. They indicate that “…. *strict adherence to any of the classification criteria, including the serological items, could lead to possible misclassification of SLE and/or delayed diagnosis…*” (57).

Their critical viewpoints rely on problems with the serological items which are problematic in the ACR, SLICC, and the EULAR/ACR classification criteria. There is neither strict definition applied to assay principles on anti-dsDNA antibodies, nor on anti-nuclear antibody (ANA) detection and impact of ANA sub-specificities. Different assay principles detect different anti-dsDNA antibodies with different unique properties (like avidities and DNA structural specificities, and with different clinical associations). This may lead to misclassification as different anti-dsDNA antibody qualities and specificities may associate with different conditions (7, 25, 45) like in malignancies and infections (45, 57).

### The SLE classification criteria from 1971 until today – an overview

For time to come, we will remain with classification criteria versions selected by expert-based procedures (10, 12, 14–16) and Delphi-panels (54). In opposition to this tradition, we now need science-based pathobiological explanations for criteria that are linked by evidence to a concise SLE pathogenesis (like anti-chromatin antibody-induced lupus nephritis associated with a defect chromatin metabolism, discussed in (18, 19, 40, 58, 59)). Before we can fulfill this need, we must define a genuine SLE- promoting process that is identifiable for clinicians. We will for now also remain with classification criteria as quasi diagnostic criteria, although they are unlinked from any known SLE etiology (4, 25, 29, 33). Reasons for this unenthusiastic view is given in the following sections that describe the spectrum of SLE classification criteria elaborated from 1917-2019. This discussion is based on definitions and arguments provided in detail above.

### The 1971 preliminary SLE classification criteria – criteria selection

The 1971 study that attempted to classify SLE, proposed 14 preliminary criteria (**Table 1**). One paradox is identifiable already when these first preliminary classification criteria was published (12). According to their intention, the first concept was “*to assemble and compare data from different sources concerning natural history, evaluation of therapy, and epidemiologic description*” (12). This ideal approach was, however, hampered by the fact that the criteria did not unequivocally identify a concise SLE diagnose or an objective causative link between the criteria and SLE. This still reflects our lack of an etiological definition of SLE.

These criteria were formulated by computer analysis of 57 symptoms, signs, and laboratory findings in 245 patients (with “unequivocal SLE”) under the care of rheumatologists in the United States and Canada (12). The classification criteria were accepted irrespective whether they appeared simultaneously, or individually one by one over non-defined time lapses. Insufficient data at the time of evaluating these records excluded further analytical criteria like anti-nuclear antibody, Coombs test, anti-DNA antibody, tissue biopsies, and direct immunofluorescence of skin biopsy specimen. These criteria influenced the next versions of SLE classification criteria.

This is demonstrated and analyzed in **Table 1** as a comparison between the 4 major SLE classification criteria versions that appeared from 1971 until 2019. Criteria in the **Table 1** marked in blue are unique solely for the classification system they are listed under. Individual criteria marked in brown are shared by some, but not all 4 criteria versions. Those written in black are shared by all 4 criteria versions. Criteria terms may be designated differently although they express the same symptoms/deviations (see footnote to **Table 1** for explanations). Thus, the 1971 preliminary classification criteria are to a large extent embedded into the subsequent classification criteria versions.

The 1971 preliminary classification criteria outlined an authoritative implementation rule: If any 4 of the 14 criteria were noticed, the patient was classified as having definite SLE. In the discussion of criteria implementation, consensus decided that immunological parameters should be excluded, since assay principles were not authorized at that time. Theoretically, any 4 out of 14 criteria may result in up to 1001 combinations (as clinical SLE phenotypes, see footnote 4 for calculation).

A problem related to investigating patients belonging to a cohort established by the SLE classification criteria is given in **Table 1**. One patient has for example lupus nephritis (Criteria # 2,3,8 and 10) for the SLE preliminary classification criteria, or criteria # 10, 18, 21 defined in the EULAR/ACR SLE classification criteria; see **Table 1** for details). Other patients are classified by other, non-nephritis criteria. These are striking differences between patients from single SLE cohorts classified by any of the classification sets described in **Table 1**. This means that within one SLE cohort, patients with and without lupus nephritis and with and without anti-dsDNA antibodies are implemented. The same classification problem appears if the 2012 SLICC or the 2019 EULAR/ACR classification criteria sets are used (see **Table 1** for details).

According to our contemporary deficient insight into SLE pathophysiology, a science-based approach to harmonize classification criteria with concise diagnostic criteria must be a prioritized task in future studies: We have to reach a definition based on pathobiological science that clarify what we mean with an “SLE diagnosis”. This definition must harmonize with description of a unique disease process(es). Today, SLE is a misperceived diagnosis with many phenotypic variants.

### Progressive revision of criteria identification in the aftermath of the 1971 preliminary classification criteria: The 1982 SLE classification criteria

The 1971 criteria can operationally be defined as symptoms/biological abnormalities expected to be present in a patient assumed to suffer from SLE. This assumption is founded on tradition, experience, consensus, and intuition – (“*you can´t explain SLE, but you understand what it is when you see it*” is a famous saying in an Anti-dsDNA antibody workshop in London in 1994), and is not established on exact science-based insight (see principal considerations in (60, 61).

In 1973, Fries and Siegel (62), on behalf of “The Diagnostic and Therapeutic Criteria Committee of the American Rheumatism Association” assembled criteria for the classification of SLE based on the 1971 preliminary SLE classification criteria version (12). During this process the committee established a database of 245 patients with unequivocal SLE (*the term “unequivocal” is basically not defined in this context*), 234 patients with rheumatoid arthritis, and 217 patients with miscellaneous non-rheumatic diseases. Data was contributed by 52 USA investigators, who were requested to submit patients with “unequivocal SLE”, and with “classic” or definite rheumatoid arthritis. Patients whose clinical course was atypical were excluded. The term “atypical” in this study lacks a firm definition based on objective parameters. It is in this context principally important to recollect that SLE is an unsolved enigma making “unequivocal” and “atypical” SLE difficult to understand or define in the aftermath of the study. This work was, in this author´s opinion, therefore inspired by ideal intentions beyond concrete rationalism. The study of Fries and Siegel (62) did not result in a formally revised SLE classification set, but data were considered in the 1982 ACR SLE classification criteria.

In 1982, Tan et al. published a revised version of the 1971 SLE classification criteria (14), cited 16337 times per April 2020). These criteria had one important advantage; implementation of laboratory testing. The essential study protocol was as follows. Patients were selected and clinical parameters were reported by investigators representing major clinics. Ten consecutive patients were to be reported by each investigator. This authorized the SLE study population to be broadly representative for SLE patients at major institutions in the USA (14). The presence or absence of each variable (of a total of 30, including all of the fourteen 1971 SLE preliminary criteria) were noted at the time of examination, *or at any time recorded in the past* (for details, see (14)). The results of this study are well known and recognized and in context of SLE classification one of the most cited studies.

The revised ACR SLE classification criteria (**Table 1**) require that at least 4 out of 11 criteria must be noted to classify SLE. The ACR criteria demonstrated higher sensitivity and specificity when compared with the 1971 criteria possibly due to implementation of laboratory parameters in the 1982 criteria (14). The 1971 criteria influenced the composition of the 1982 ACR criteria (**Table 1**) and they shared many of the criteria. They both establish cohorts that theoretically described patients with multiple different clinical SLE phenotypes (1001 and 330 theoretically different criteria combinations (here: synonymous with phenotypes) for the 1971 and 1982 classification criteria, respectively).

The same problem with respect to heterogenic SLE cohorts appears with the 1982 ACR criteria as with the 1971 criteria: One patient may have lupus nephritis and anti-dsDNA antibodies, the other patient may have other criteria. Both are classified as having SLE. This difference challenges the dogmatic characterization of ACR-derived cohorts as implementing a disease entity (see above). One theoretical aspect of the 1982 study is relevant for all the classification criteria versions: The heterogenous SLE cohorts hinders studies ideally aimed to studies of homogenous patient cohorts.

### The next milestone: The 2012 SLICC SLE classification criteria

The 2012 SLICC SLE classification criteria did not change the basic problems described above. In their report (16) Petri et al. stated that *“The Systemic Lupus International Collaborating Clinics (SLICC) group revised and validated the American College of Rheumatology (ACR) systemic lupus erythematosus classification criteria in order to improve clinical relevance, meet stringent methodology requirements, and incorporate new knowledge regarding the immunology on SLE. The classification criteria were derived from a set of 702 expert-rated scenarios. Recursive partitioning was used to derive an initial rule that was simplified and refined based on SLICC physician consensus”.*

The final SLICC classification criteria contained 17 criteria (11 clinical and 6 immunological ones, **Table 1**). The final validation was performed by comparing the final 17 SLICC criteria with the 11 modified ACR 1997 criteria (15). The SLICC criteria performed well with higher sensitivity, but lower specificity, and had a lower (but still a high) number of misclassified cases (74 versus 62 for the 1997 revised ACR criteria and the final SLICC criteria, respectively). The final instruction for the SLICC criteria is that 4 criteria is required out of 17. At least one clinical and at least one immunological criterion have to be identified to classify a patient to have SLE. One important exception from this rule is that lupus nephritis with anti-DNA antibodies is sufficient to fully classify SLE. Therefore, lupus nephritis and anti-dsDNA antibodies serve as diagnostic *and* classification criteria, although the idiom “diagnostic criteria” is not a formally accepted term for SLE.

### Impact of the SLICC SLE classification criteria – intentions and limitations

What can we extract from the SLICC criteria, and for what purpose can the SLICC criteria be fruitfully implemented in basic studies of SLE? Although the SLICC criteria were validated as superior to the ACR criteria, the SLICC criteria was influenced by the guidelines defined in the 1971 preliminary classification criteria, and by the investigators´ subjective experience and diagnostic traditions. The SLICC classification criteria therefore inherit the impact of the former criteria versions (see **Table 1** for comparisons), but the criteria inherit also many of the same problems, including the fact that also the SLICC criteria promote cohorts which are substantially heterogeneous with respect to phenotypic diversity.

According to the criteria, the clinical phenotypes determined according to the SLICC rules may or may not comprise serious SLE criteria like lupus nephritis and anti-DNA antibodies. This simple fact may be a remarkable argument to reconsider implementation of the SLICC criteria in studies of basic SLE processes accounting for causal *etiology* and *pathogenesis* of the syndrome. Simply expressed, disparate SLE conditions may be linked to unrelated etiologies, genetics and pathophysiology, but not to one concise SLE diagnosis. We still lack a concise diagnostic marker for SLE.

For example in RA, affection of joints is the central elements (63), in primary Sjögren syndrome, the sicca symptom is essential (64). In something that could be denoted *primary SLE*, lupus nephritis (19, 25) and dermatitis (20, 21) could be evidentiary for SLE, and used as criteria for homogeneous cohorts.

### The 2019 EULAR/ACR SLE classification criteria

The most recent effort to refine and improve SLE classification criteria was supported by the European League Against Rheumatism (EULAR) and the American College of Rheumatology (ACR (10, 26, 56),. Initially, Aringer et al. performed a systematic review and meta-regression analysis for two purposes: *I*, to evaluate if ANA could serve as an authoritative entry criterion, and *ii.* to generate new and more precise arrays of classification criteria.

In this context, Leuchten et al. published in 2018 an analysis of data aimed to describe performance of antinuclear antibodies for classifying SLE, and if ANA justified a position as a mandatory entry criterion for SLE cohorts (65). Only screening methods for ANA were emphasized. Notably, this approach ignored a discussion on which ANA sub-specificities are covered by the screening assays. Furthermore, the potential diagnostic and pathogenic impact of ANA sub-specificities were not given attention (10, 29). Here the clinicians are in conflict with the immunologists. According to concise theoretical insight, ANA are widely detected in infections (66), cancers ( (67), see a discussion in (25) and references therein), autoimmune diseases/syndromes (68), and after ingestions of certain drugs (69). Their manifestations in non-SLE contexts are substantial. On the other hand, Choi et al. observed that 6.2% of SLE patients were ANA negative (70). It may thus appear as problematic that the 2019 EULAR/ACR classification criteria for SLE recommend positive but unspecified ANA at least only once as an obligatory entry criterion for SLE classification.

Criteria generation for the new version of classification criteria was performed through an international Delphi exercise including criteria embedded in previous classification criteria versions, an early diagnosed patient cohort, and a patient survey (see details in Aringer et al. (10), and Delphi panel details in (54)). Criteria reduction followed Delphi-panels and nominal group technique exercises, while criteria definition and weighting were based on criteria performance and on results of a multi-criteria decision analyses. Schmajuk et al. opened for the view that *“1* (one) *organ system would be sufficient for classifying SLE*” (54). This may be beneficial for investigating SLE as a homogenous syndrome dominated by lupus nephritis. If this suggestion refers to *any* organ system it may be problematic and transform the cohort into being heterogeneous and less valid.

The final version of the EULAR/ACR SLE classification criteria contains 21 weighted criteria (**Table 1**) organized into 10 domains (10). Occurrence of a criterion on one occasion is sufficient. Definite SLE classification requires at least one clinical criterion and weighted criteria ≥ 10 points, and a positive ANA entry criterion. The unbiased combination of the 21 criteria has as a clear consequence a manifold of disparate SLE conditions, as is the case for all the SLE classification criteria over the last 50 years (see details in **Table 1**).

Sensitivity and specificity of the EULAR/ACR criteria compared well with the 1997 revised ACR and the 2012 SLICC criteria. This may be anticipated due to the fact that many of the 1971 criteria are embedded and shared in the 1982 ACR (14), the 1997 revised ACR (15), the 2012 SLICC (16), and most recently in the 2019 ACR/EULAR SLE classification criteria ( (10), see **Table 1** for details).

## General comments on SLE classification criteria

It may be contemplated that the many clinical phenotypes that emerge if using any of the SLE classification criteria versions may reflect an “iatrogenic” impact explaining the disparate categories of the disease. There is no radical evolution of the classification criteria and their use in defining SLE patients - and there is no implementation of diagnostic criteria - over the last 50 years, except for anti-dsDNA antibodies and lupus nephritis as proclaimed in the SLICC and the EULAR/ACR criteria, where anti-dsDNA antibodies and nephritis together are weighted to 10 points in the latter criteria. Maybe we have the knowledge sufficient to understand what SLE is, but do we still lack the courage to split the syndrome into “hot SLE”, e.g. characterized by autoantibody-driven lupus nephritis, and a less distinct category like “overlapping lupus-like disorders”.

One theoretical and logic approach would be studies of SLE cohorts exclusively defined as “hot SLE” defined as lupus nephritis induced by anti-dsDNA antibodies (see a discussion on the impact of anti-DNA antibodies in (45, 71, 72)). An analogous approach could be to study a cohort defined solely by the butterfly exanthema with a positive lupus band test (73, 74). The latter would be an interesting study also in an historical context since the history of SLE starts with the antique narrative of a serious cutaneous disease – the “lupus” (see aspects of the history of lupus in antiquity up to contemporary times (75–78)). From these reflections, the contour of a distinction between “SLE” and “lupus-like” diseases can be comprehended.

### A paradox: Monogenic SLE is defined as “lupus-like” while incoherent criteria classify SLE as “unequivocal SLE” or simply SLE!

In concrete words, SLE is still defined by criteria that are limited to be conceptual and informal in nature: There is no unifying impact of classification criteria on our insight into etiology and pathogenesis that *define* the human variants of SLE, except for some rare gene deficiencies (see e.g (79–81).).

The gene-deficient “lupus-like” diseases are often pauci-symptomatic (80, 81) with single pathogenic pathways typically centered around autoimmunity like production of anti-dsDNA antibodies, development of antibody-dependent nephritis and skin affections. For example, a null mutation of DNase 1L3 was recently described as the etiological component in familiar SLE (42). This was experimentally confirmed in DNASE 1L3-deficient mice, in which a lupus-like disease characterized by autoantibodies to DNA and chromatin constituents and development of lupus nephritis (39, 40). It is difficult to understand that monogenic SLE often is denoted “lupus-like” while being instigated by single gene defects that results in objective SLE-related criteria (see e.g. references in (79, 80)). SLE classified by criteria are simply and authoritatively denoted (unequivocal) “SLE” although definitively being characterized as “enigmatic” (discussed in (4, 25, 72)).

Gene deficiencies as concise causes of “lupus-like” disease variants may theoretically shed light on the problem “one defect (here: synonymous with one etiological origin) – several diversified symptoms” as a term for lupus-like variants. For example, a majority of patients with C1q deficiency develop a lupus-like phenotype including clinical symptoms such as photosensitivity, skin rash, nephritis, oral ulcers, and arthritis. Similar biologically confined spectra of symptoms have been observed in other monogenic defects (see e.g. concise discussions in (79–82). In this context, one identified cause for SLE bring several SLE classification criteria to the scene. Maybe monogenic SLE can provide us with the information necessary to develop *SLE diagnostic criteria*. A discussion by Touma et al. harmonizes well with thoughts presented here, that some criteria are linked during chains of specific disease processes (83) exemplified in (84) for the link between loss of renal DNase 1, anti-dsDNA antibodies and nephritis). Monogenic SLE or murine lupus-like conditions may be valid models to study the origin and progression of complicating spectra of SLE classification criteria. This approach may examine if all listed SLE classification criteria indeed are linked to an autoimmune origin with a subsequent corresponding broad autoimmune pathophysiology.

## Concluding remarks

The conclusions of this study are not very optimistic, and can be summarized as follows:

- SLE classification criteria have been practically ascribed the function of diagnostic criteria – whether we like it or not.
- The classification criteria do not define SLE as one disease with one etiology, and with one emerging clinical phenotype.
- The classification criteria are far from being optimal to select SLE patients for basic studies of a unifying etiology and pathogenesis
- We now need new paradigms to promote a rational and insightful definition of SLE – a definition based on evidence-based facts, not by “democratic” processes like Delphi panels.
- In sum: We need to develop concise diagnostic criteria that define a homogeneous SLE cohort as a starting point to describe the principal origin of the syndrome, and to separate the primary from secondary disease processes.

We are still far from implementing precision diagnostics of SLE. We have to realize that we classify a syndrome that lacks a science-based definition. Today, classification criteria as strategical instruments in SLE research are too imprecise if the aim is more concise studies intended to reduce the enigmatic character of SLE.

## Data availability statement

The original contributions presented in the study are included in the article/supplementary material. Further inquiries can be directed to the corresponding author.

## Author contributions

I, OPR, am the sole author of this manuscript. I am responsible for content, arguments and interpretations. I am responsible for the decision to submit this manuscript to Frontiers in Immunology.

## Acknowledgments

I am thankful to Marco Radic, University of Tennessee Health Science Center, for continuous critical discussions related to the controversies in the scientific field of SLE. I am thankful to Gunnar Rekvig, UiT-The arctic University of Norway, for textual and logic improvements during fulfillment of this manuscript. A warm thank to Rod Wolstenholme, UiT-The arctic University of Norway, who patiently discussed data and their presentation in this text.

## Conflict of interest

The author declares that the research was conducted in the absence of any commercial or financial relationships that could be construed as a potential conflict of interest.

## Publisher’s note

All claims expressed in this article are solely those of the authors and do not necessarily represent those of their affiliated organizations, or those of the publisher, the editors and the reviewers. Any product that may be evaluated in this article, or claim that may be made by its manufacturer, is not guaranteed or endorsed by the publisher.

## Footnotes

1 Merriam-Webster.com Dictionary, https://www.merriam-webster.com/dictionary/criterion

2 In this context, a clinical phenotype is defined as an observed phenomenological presentation of a disease without any designation to an etiology or a pathophysiologic process. This is in the present context an important distinction, as a syndrome like SLE may present different phenotypes linked to various separate disease mechanisms, all for the time being denoted by the unifying term “SLE”. Consequently, the term “clinical phenotype” or just “phenotype” is here used to described appearance of a disease in a given individual or cohort.

3 https://www.merriam-webster.com/dictionary/entity

4 Merriam-Webster

5 Abstract syndrome means here pathophysiological processes accounting for apparently non-coherent and inexplicit composite clinical symptoms and biological aberrances – the latter called criteria. *These are included by intuitive and statistical more than biologically relevant analyzes of SLE cohorts*.

7 For example, anti-dsDNA antibodies may by themselves be clinical epiphenomena, but in context of lupus nephritis, their presence may put a focus on impaired chromatin metabolism in the kidney, leaving chromatin fragments exposed in glomeruli as targets for the antibodies. In this context, lupus nephritis is basically not an autoimmune organ disease, but a chromatin metabolic disease that may induce secondary clinically overt immunopathologies (35;36).
